# Retraction: Intraosseous Calcaneal Lipoma Misdiagnosed as Plantar Fasciitis: An Orthopedic Case From Family Practice

**DOI:** 10.7759/cureus.r121

**Published:** 2024-01-25

**Authors:** Alaa M Alnooh, Bashayer F Al Furaikh, Abdullah M Alaithan, Abdulaziz K Halawani, Mohammed F Al-Khalifah, Mohammad O Abu Zahirah, Mutasim H Alhasani, Abdullmgeed A Asiri, Aqeel H Alrashid, Eyad H Alfaqih, Mohammed A Khair, Seddiqa S Naiser, Yaqeen S Alkamel, Abdulaziz A Al Mohammed, Faisal Al-Hawaj

**Affiliations:** 1 College of Medicine, Alfaisal University, Riyadh, SAU; 2 College of Medicine, King Faisal University, Hofuf, SAU; 3 General Practice, King Abdullah Medical City, Riyadh, SAU; 4 College of Medicine, Umm Al-Qura University, Mecca, SAU; 5 College of Medicine, King Saud University, Riyadh, SAU; 6 College of Medicine, King Khalid University, Abha, SAU; 7 College of Medicine, Taif University, Taif, SAU; 8 College of Medicine, King Saud Bin Abdulaziz University For Health Sciences, Riyadh, SAU; 9 College of Medicine, Xi’an Jiaotong University, Xi'an, CHN; 10 College of Medicine, Imam Abdulrahman Bin Faisal University, Dammam, SAU

The Editors-in-Chief have retracted this article. Concerns were raised regarding the identity of the authors on this article. Specifically, Faisal Alhaway and Malak Shammari have stated that they were added to this article without their knowledge or approval. The identity of the other authors could also not be verified. As the appropriate authorship of this work cannot be established, the Editors-in-Chief no longer have confidence in the results and conclusions of this article.

